# Supplementary material for: Gli1 identifies osteogenic progenitors for bone formation and fracture repair
Source: Nat Commun. 2017 Dec 11;8:2043. doi: 10.1038/s41467-017-02171-2 (PMC5725597; doi:10.1038/s41467-017-02171-2)
Supplement: Supplementary file 1 — Supplementary Information [file 41467_2017_2171_MOESM1_ESM.pdf]

Supplementary Figure 1

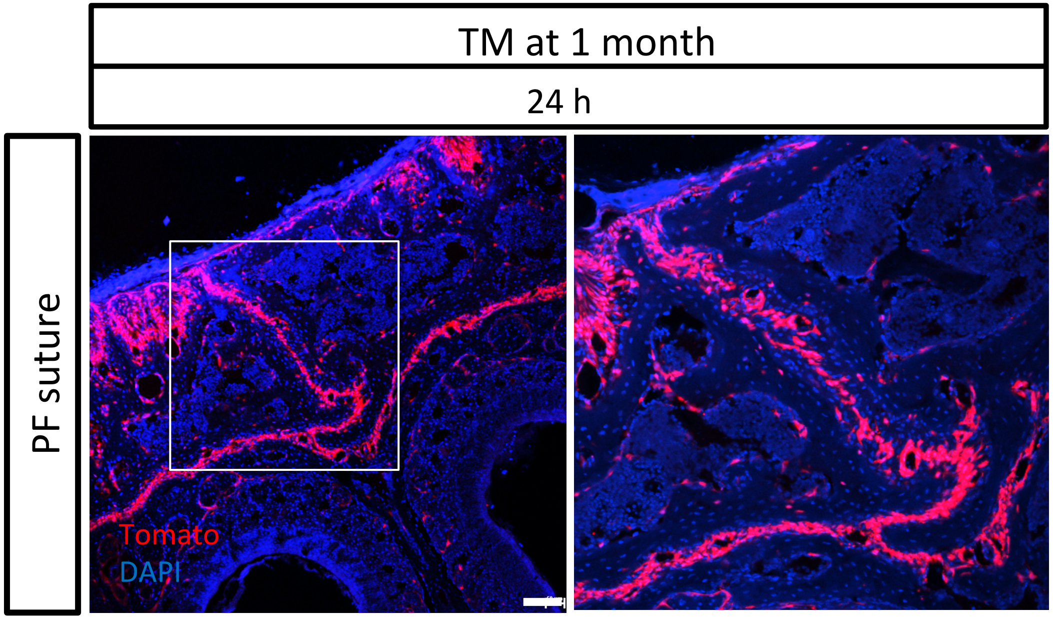

Supplementary Figure 1. Gli1$^+$ cells in the posterior frontal (PF) suture.

Gli1-CreER^{T2}; Ai9 mice were administered TM at one month of age and harvested 24 hrs later.

Scale bar: 100 μm. Boxed region shown at a higher magnification to the right.

Supplementary Figure2

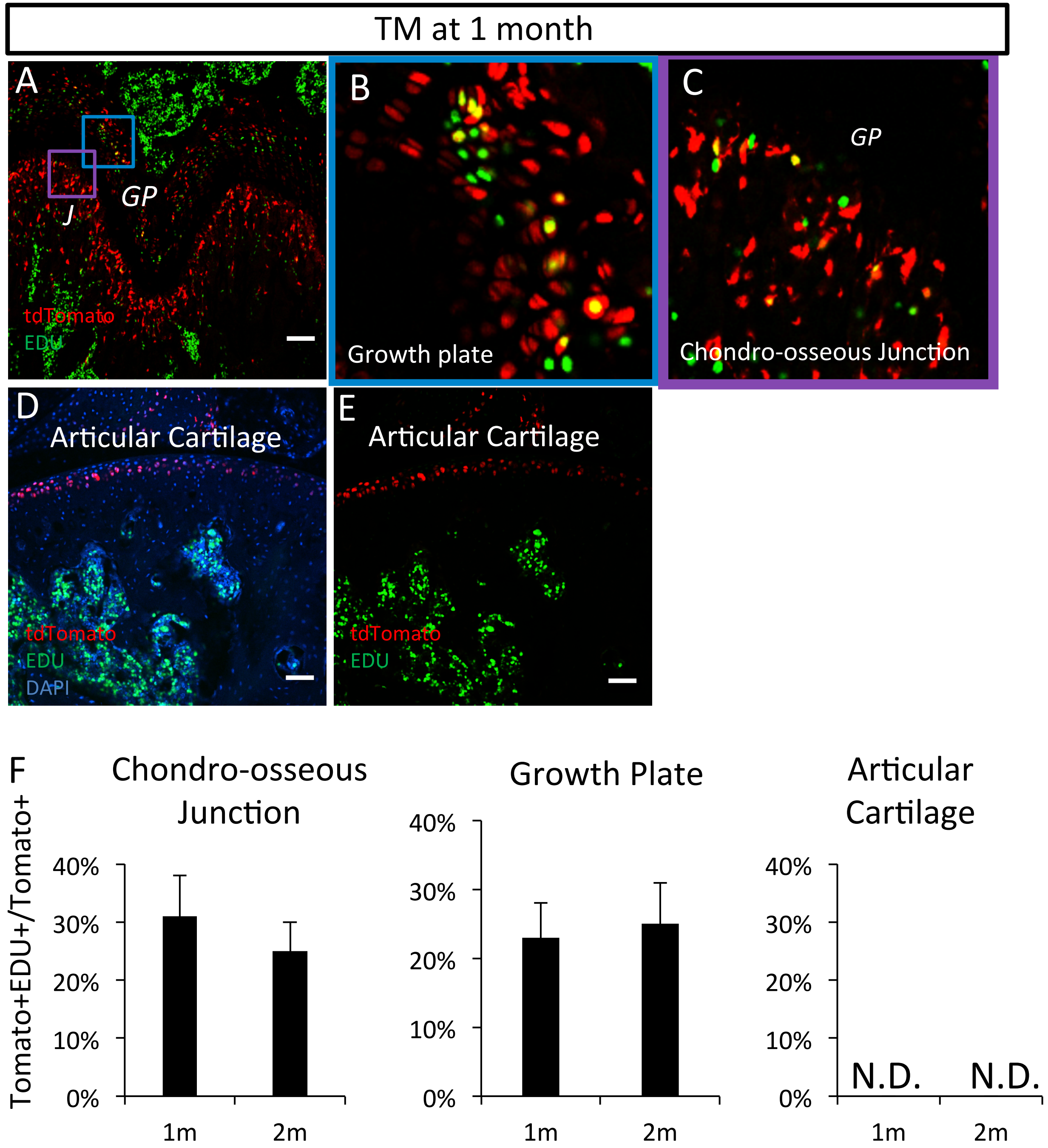

Supplementary Figure 2. Proliferation assays of different Gli1$^+$ cell populations.

Gli1-CreER^{T2}; Ai9 mice were administered TM at one month of age and injected EdU 24hrs later. Mice were sacrificed at 4 hrs after EdU injection. Scale bars: 100 µm.

(A) Fluorescence images of a longitudinal section through the distal end of femur. Red: tdTomato; green: EdU immunostaining; yellow: co-localization. Boxed areas of the growth plate ("*GP*") or the chondro-osseous junction ("*J*") are shown to the right at a higher magnification (B, C).

(D, E) Fluorescence images of a section through the distal femur containing the articular cartilage with (D) or without (E) the DAPI signal (blue). Note no co-localization of tdTomato and EdU among the articular chondrocytes.

(F) EdU labeling index among different Gli1$^+$ populations (% tdTomato$^+$EdU$^+$/tdTomato$^+$) in mice at one (1m) or two (2m) months of age with TM at 1 month or 2 months. N.D.: not detectable. Labeling percentage derived from one representative section each of three mice at each age.

Supplementary Figure 3

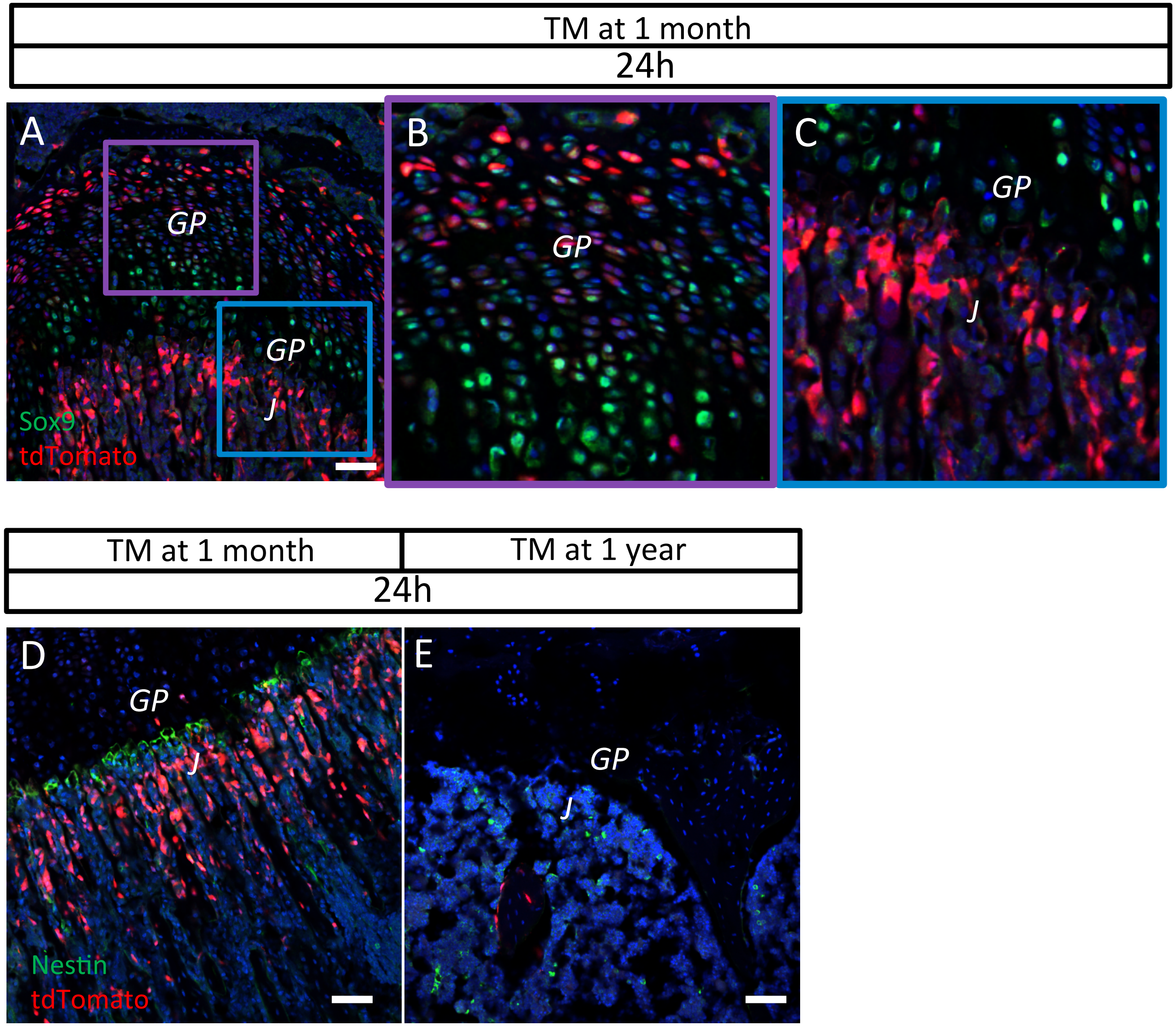

Supplementary Figure 3 MMP does not express Sox9 or nestin.

Gli1-CreER^{T2}; Ai9 mice were administered TM at one month (A-D) or one year (E) of age and harvested 24 hrs later.

(A) Sox9 immunostaining of a section through the distal growth plate of a femur. Boxed areas were shown to the right (B, C). Note that Sox9 (green) and tdTomato (red) co-localize (yellow) in growth plate ("*GP*") chondrocytes but not MMP at the chondro-osseous junction ("*J*").

(D, E) Nestin immunostaining of sections through the distal growth plate of femurs. Note little to no overlap between nestin (green) and tdTomato (red). Few MMP (red) remained in one-year-old mice (E). Scale bar: 100 µm.
